# Comparing the sleep benefits of Nature-Based Interventions: Evidence from a Bayesian Network meta-analysis

**DOI:** 10.1016/j.isci.2026.115513

**Published:** 2026-03-28

**Authors:** Cong Ma, Aamir Mehmood Shah, Wei Chen, Zongsheng Li, Yi Peng, Ruohan Wu, Xinyu Du, Hao Li, Bingyang Lv, Shiliang Liu, Qibing Chen

**Affiliations:** 1 College of Landscape Architecture, Sichuan Agricultural University, Chengdu 611130, China; 2 Department of Architecture and Design (DIAP), Sapienza University of Rome, 00185 Rome, Italy

**Keywords:** health sciences, environmental health

## Abstract

Sleep health represents a growing global public health concern, underscoring the urgent need for scalable non-pharmacological interventions. We conducted a Bayesian network meta-analysis of randomized controlled trials (RCTs) to synthesize comparative evidence on sensory-oriented nature-based interventions (NBIs) for sleep health in adults. Across the evidence network, all intervention categories consistently outperformed conventional care, whereas between-modality differences were generally modest. This suggests that the therapeutic benefits stem from a shared effect of nature exposure, rather than a single dominant mechanistic pathway. Robustness analyses confirmed the stability and reliability of the comparative effect estimates. These findings provide an integrative synthesis of randomized evidence and support NBIs as accessible, scalable strategies to improve sleep health. They also underscore the critical need for larger, more rigorously designed RCTs to refine estimates of the comparative effectiveness of different NBI modalities.

## Highlights

• Nature-based interventions consistently improved adult sleep health across trials
• Comparative benefits across sensory-oriented interventions were generally modest
• All active nature-based approaches outperformed conventional care
• Bayesian evidence synthesis supports scalable, non-pharmacological sleep strategies

## Graphical abstract

**Figure:**
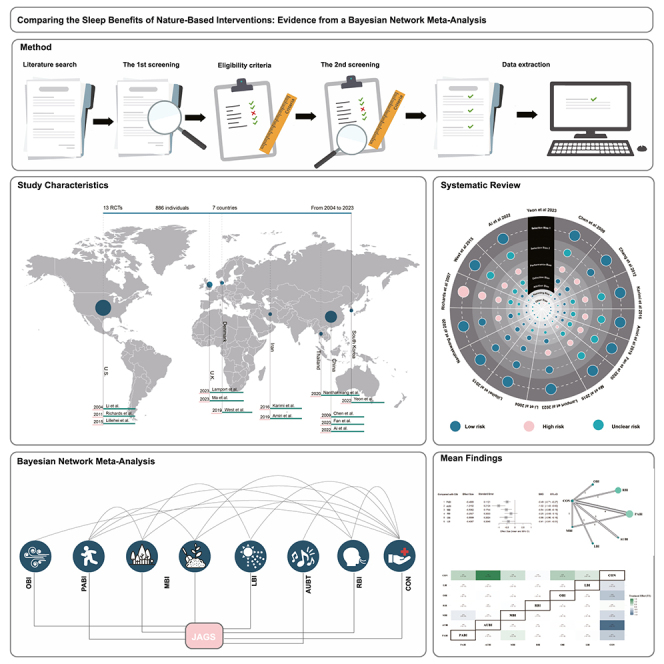

## Introduction

Sleep health problems, encompassing both sleep disturbances and clinically defined sleep disorders, represent a substantial and growing public health burden worldwid.^1^^,^^2^^,^^3^^,^^4^ Recent epidemiological estimates indicate that approximately 852 million adults worldwide are affected by insomnia, corresponding to a global prevalence of 16.2%, of whom an estimated 415 million experience severe insomnia.^5^ These conditions contribute to increased morbidity, reduced quality of life, and substantial healthcare costs, thereby underscoring the urgency of identifying effective and scalable interventions.^6^^,^^7^ In light of the magnitude of this public health challenge, there has been an escalating focus on non-pharmacological and environmental approaches that have the potential to promote sleep health in a safe and scalable manner. Nature exposure entails direct multisensory engagement with natural environments through visual, auditory, olfactory, and tactile channels.^8^^,^^9^^,^^10^^,^^11^^,^^12^^,^^13^^,^^14^^,^^15^ This exposure spans both active and passive interactions with natural settings (including urban green spaces and coastal areas) and fosters psychological connectedness to nature that may mediate its restorative benefits.^16^^,^^17^^,^^18^^,^^19^^,^^20^^,^^21^^,^^22^^,^^23^^,^^24^^,^^25^ Over the past several years, the link between nature exposure and health has attracted considerable attention globally, underscoring its significance across different populations and demographic groups.^26^^,^^27^^,^^28^^,^^29^^,^^30^^,^^31^^,^^32^^,^^33^^,^^34^^,^^35^

Among the many health benefits associated with exposure to nature, the impact on sleep is particularly significant.^36^^,^^37^^,^^38^^,^^39^^,^^40^^,^^41^ In fact, sleep is widely acknowledged as a crucial cornerstone of overall physiological and psychological health.^42^^,^^43^^,^^44^^,^^45^^,^^46^^,^^47^ Growing evidence suggests that exposure to natural settings, such as green landscapes and aquatic environments, could have a beneficial effect on sleep health, supporting both psychological and physiological recovery.^48^^,^^49^^,^^50^^,^^51^^,^^52^^,^^53^^,^^54^^,^^55^^,^^56^^,^^57^^,^^58^ These results emphasize the substantial public health advantages of engaging with natural environments, particularly in improving sleep health and overall wellness.^59^^,^^60^ Exploring how exposure to natural environments impacts sleep is vital, as it contributes not only to improving individual health and well-being but also to reinforcing societal and economic resilience.^61^^,^^62^

The influence of nature exposure on sleep has been extensively investigated, with researchers identifying a range of potential interventions to address sleep issues. As such, nature exposure is increasingly recognized as a source of practical, scalable solutions for sleep enhancement.^63^^,^^64^^,^^65^^,^^66^ For example, studies indicate that interaction with natural landscapes (including forests, greenways, parks, and lakes) is associated with significant improvements in sleep health.^67^^,^^68^^,^^69^^,^^70^^,^^71^^,^^72^^,^^73^ However, substantial gaps remain in understanding the conditions under which green environments influence sleep outcomes, particularly given the variability across individuals and environmental contexts.^74^^,^^75^^,^^76^^,^^77^^,^^78^^,^^79^^,^^80^ Beyond general nature exposure, many studies have identified more structured forms of environmental contact, commonly referred to as nature-based interventions (NBIs). NBIs are defined as planned or semi-structured programs that intentionally integrate exposure to natural settings, including forests, parks, and other green environments, with the aim of promoting specific health outcomes.^81^^,^^82^ In the context of this review, we focus specifically on NBIs rather than general nature exposure. Conceptually, NBIs can be viewed as structured forms of nature exposure that operate through one or more dominant sensory and perceptual pathways. NBIs offer clearer operational definitions, greater comparability across studies, and more standardized intervention components, which makes them more suitable for evidence synthesis through network meta-analysis.

Earlier studies have shed light on how interactions with natural environments influence sleep health.^31^^,^^83^^,^^84^^,^^85^^,^^86^^,^^87^^,^^88^^,^^89^^,^^90^ Nevertheless, many of these publications primarily employ observational designs and face geographical constraints, compromising the generalizability of their findings.^91^^,^^92^^,^^93^^,^^94^^,^^95^^,^^96^^,^^97^ Moreover, the heterogeneity in methodologies and outcome measures across studies has made it challenging to synthesize robust and widely applicable conclusions.^64^^,^^98^^,^^99^^,^^100^ Bayesian network meta-analysis (BNMA) enables the simultaneous comparison of multiple interventions by integrating both direct and indirect evidence within a single analytical framework.^101^ Unlike traditional pairwise meta-analysis, BNMA integrates both direct and indirect comparisons, quantifies uncertainty in treatment rankings, and is well-suited for evidence bases that are small, diverse, or structurally imbalanced. These features make BNMA particularly appropriate for NBI research, where intervention formats, durations, and populations vary widely across studies.^102^^,^^103^

The present study aims to conduct a comparative evaluation of the effects of diverse NBIs on sleep outcomes, using a unified BNMA framework. Specifically, we seek to address three objectives: (1) to assess and compare the effectiveness of different categories of NBIs classified according to their primary sensory or experiential modality, including physical activity-based interventions (PABIs), auditory-based interventions (AUBIs), olfactory-based interventions (OBIs), light-based interventions (LBIs), respiratory-based interventions (RBIs), and multisensory-based interventions (MBIs); (2) to assess whether multisensory and single-modality NBIs are associated with greater improvements in sleep outcomes compared with conventional care (CON); and (3) to apply BNMA to synthesize both direct and indirect evidence, probabilistically rank all NBI categories, and quantify uncertainty in their relative treatment effects.

## Results

### Characteristics of included studies

A total of 13 RCTs published between 2004 and 2023 were included in the analysis, comprising a total of 886 adult participants, with balanced allocation between intervention and control groups. The included trials evaluated a range of NBIs classified according to their primary sensory or experiential modality, including PABI, AUBI, OBI, LBI, RBI, and MBI. The control conditions primarily involved CON. Only one study included a head-to-head comparison between two active NBI modalities (Table 1). Across studies, intervention durations exhibited significant variability, ranging from brief exposures lasting several days to more extensive interventions extending up to six months. The variability in intervention modality, exposure duration, participant age, and comparator type constituted the primary sources of clinical and methodological heterogeneity in the included evidence. A comprehensive array of study-level information is delineated in Table 1.

**Table 1:** Basic characteristics of included randomized controlled trials (x ± s)

Study	Time	Country	Sample size (Male/Female)		Mean age (year)		Intervention		Duration	Main Outcome
			T	C	T	C	T	C		
Li et al.^104^	2004	U.S	M:10 Fe:52	M: 12 F:44	75.3 ± 7.8	75.4 ± 7.8	PABI	CON	24 weeks	PSQI
Chen et al.^105^	2009	China	M: 10 F:52	M: 25 F:41	65.77 ± 4.32	72.42 ± 6.04	PABI	CON	6 months	PSQI
Richards et al.^106^	2011	U.S.	M: 21 F:34	M: 17 F:26	83.2 ± 6.5	82.7 ± 7.2	PABI	CON	7 weeks	PSQI
Lillehei et al.^107^	2015	U.S	M:14 F:15	M:10 F:29	20.9 ± 2.3	22.1 ± 4.8	OBI	CON	5 nights	PSQI
Karimi et al.^108^	2016	Iran	M: 23 F:0	M: 23 F:0	67.49 ± 4.28	66.82 ± 3.84	PABI	CON	2 months	PSQI
West et al.^109^	2019	Denmark	M:24 F:15	M: 20 F:12	72.7 ± 8.3	72.8 ± 6.1	LBI	CON	45 days (T)/34 days (C)	PSQI
Amiri et al.^110^	2019	Iran	M: 15 F:15	M: 0 F:0	27.48 ± 3.02	27.12 ± 2.12	AUBI	CON	6 sessions	PSQI
Fan et al.^111^	2020	China	M:12 F:55	M: 22 F:50	70.3 ± 5.7	71.8 ± 6.7	PABI	CON	24 weeks	PSQI
Nanthakwang et al.^112^	2020	Thailand	M: 8 F:22	M: 6 F:23	70.43 ± 7.06	70.10 ± 8.33	AUBI	CON	8 weeks	PSQI
Ai et al.^113^	2022	China	M: 2 F:10	M: 2 F:10	54.17 ± 6.70	55.25 ± 8.57	PABI	CON	12 weeks	PSQI
Yeon et al.^114^	2022	South Korea	M:7 F:15	M:1 F:24	37.8 ± 10.3	38.9 ± 10.5	MBI	CON	6 weeks	PSQI
Lamport et al.^115^	2023	U.K.	M:7 F:22	M:7 F:22	35.1 ± 9.8	34.9 ± 10.2	RBI	CON	2 weeks	PSQI
Ma et al.^116^	2023	U.K.	–	–	23.6 ± 2.23	23.6 ± 2.23	MBI	PABI	7 days	PSQI

T, intervention group; C, control group; PABI, physical activity-based interventions; AUBI, auditory-based interventions; OBI, olfactory-based interventions; LBI, light-based interventions; RBI, respiratory-based interventions; MBI, multisensory-based interventions; CON, conventional care; PSQI, Pittsburgh Sleep Quality Index.

### Risk of bias

**Figure 1:**
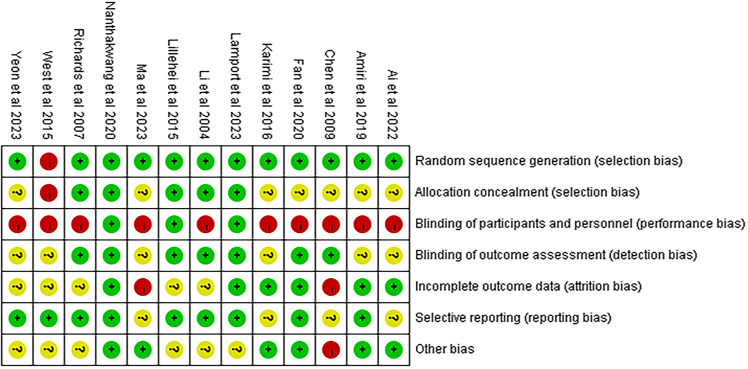
Risk of bias assessment of included randomized controlled trials Risk of bias for each included randomized controlled trial, assessed using the Cochrane Risk of Bias 2 (RoB 2) tool. Each column represents a bias domain, and colors indicate judgments of low risk, some concerns, or high risk of bias.

The risk of bias assessment across the 13 included RCTs indicated an overall moderate methodological quality, with substantial variation across bias domains (Figure 1). The 13 studies included in the analysis were found to have a low risk of bias in random sequence generation, suggesting that randomization procedures were adequately implemented across the evidence base. For allocation concealment, 6 studies (46.2%) were rated as low risk, 4 studies (30.8%) were rated as having unclear risk due to insufficient reporting, and 3 studies (23.1%) were rated as high risk, reflecting substantial limitations in preventing foreknowledge of group allocation. The most critical methodological concern pertained to performance bias. A mere 3 studies (23.1%) were classified as low risk, while the remaining 10 studies (76.9%) were deemed to be at high risk. This disparity is primarily attributable to the inherent challenge of ensuring participant and personnel blinding in non-pharmacological NBIs. In contrast, blinding of outcome assessment was more frequently reported: 7 studies (53.8%) were rated as low risk for this domain, while the remaining 6 studies (46.2%) were rated as unclear risk, due to a lack of explicit descriptions of blinding procedures. With regard to incomplete outcome data, the majority of trials exhibited acceptable data completeness. The 8 studies (61.5%) were adjudged to have a low risk of attrition bias, 4 studies (30.8%) had an unclear risk classification due to limited reporting on missing data handling, and one study (7.7%) was rated as high risk. The risk of selective reporting bias was minimal across the included studies. Ten studies (76.9%) were evaluated as low risk, while 3 studies (23.1%) were designated as unclear risk due to the unavailability of accessible study protocols or trial registrations. Finally, other sources of bias demonstrated equivocal results. The 6 studies (46.2%) were classified as low risk, 5 studies (38.5%) were deemed unclear risk, and 2 studies (15.4%) were considered high risk, primarily due to baseline imbalances, clustering effects not fully accounted for, or contextual and environmental confounders.

### Comparative structure and evidence distribution

**Figure 2:**
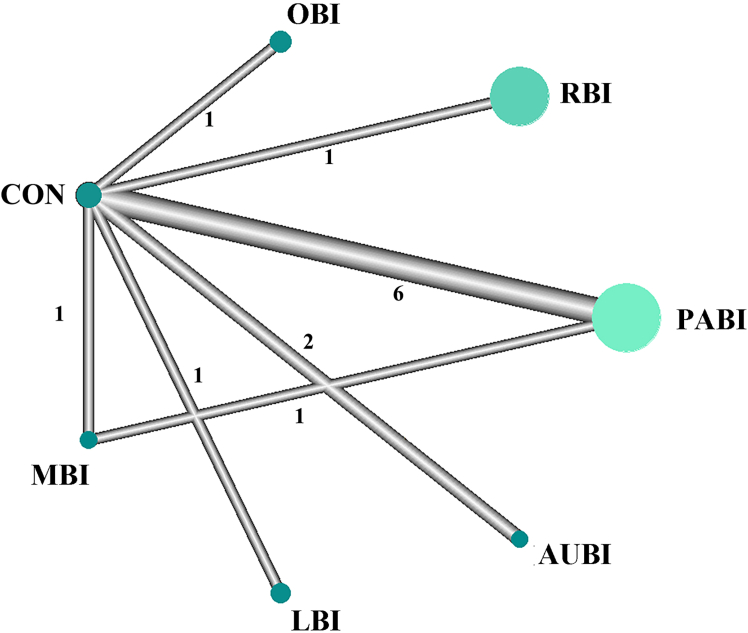
Network structure of treatment comparisons in the Bayesian network meta-analysis This network plot illustrates the evidence structure of the included randomized controlled trials. Nodes represent intervention categories, with node size proportional to the total cumulative sample size for each intervention. Edges represent direct comparisons between two interventions, with line thickness proportional to the number of trials contributing to each comparison. Abbreviations: PABI, physical activity-based interventions; AUBI, auditory-based interventions; OBI, olfactory-based interventions; LBI, light-based interventions; RBI, respiratory-based interventions; MBI, multisensory-based interventions; CON, conventional care.

The network was derived from 13 RCTs, with direct comparisons predominantly conducted between each NBI category and CON (Figure 2). The PABI vs. CON comparison was the most frequently studied contrast, supported by six trials, whereas all other NBI categories were each linked to CON by a single study. However, it should be noted that only a single head-to-head comparison between active interventions (MBI vs. PABI) was available. Consequently, CON functioned as the central anchor node, thereby enabling indirect comparisons across NBI categories. The node size reflected the cumulative sample size, with PABI contributing the largest share of evidence, while edge thickness corresponded to the number of direct comparisons. The network manifested a star-shaped yet interconnected configuration, offering ample direct and indirect evidence to substantiate BNMA across all intervention categories.

### Comparative effects of interventions on sleep quality

**Figure 3:**
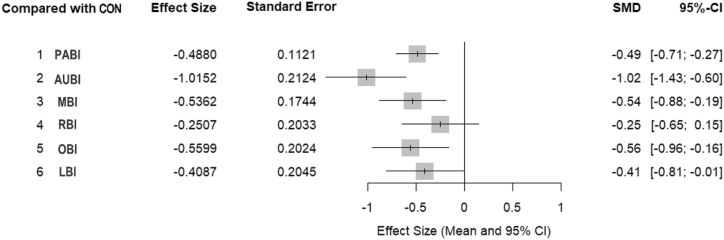
Forest plot of standardized mean differences for interventions versus conventional treatment This forest plot presents pooled standardized mean differences (SMDs) and corresponding 95% credible intervals (CrIs) estimated from the Bayesian network meta-analysis. Negative SMD values indicate improvements in sleep quality relative to conventional care (CON). All estimates are derived from a random-effects model. Total included studies: *n* = 13. Abbreviations: PABI, physical activity-based interventions; AUBI, auditory-based interventions; OBI, olfactory-based interventions; LBI, light-based interventions; RBI, respiratory-based interventions; MBI, multisensory-based interventions; CON, conventional care.

**Figure 4:**
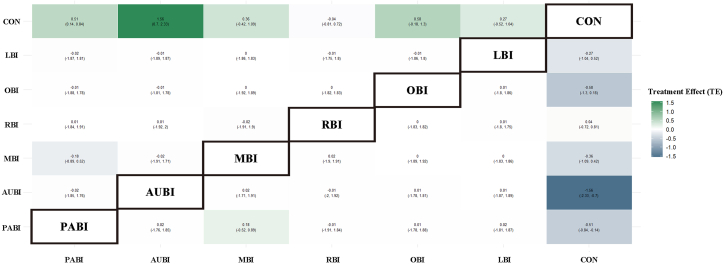
Effect size matrix of pairwise comparisons with 95% credible intervals This matrix of relative treatment effects presents standardized mean differences and 95% credible intervals (CrIs) for all pairwise comparisons between intervention categories. Cells below the diagonal show effect estimates, while cells above the diagonal present the corresponding 95% CrIs. Negative values indicate effects favoring the column-defining intervention.

Pooled standardized mean differences (SMDs) and corresponding 95% credible intervals (CrIs) for all intervention categories relative to CON are presented in Figure 3. The estimates were obtained from a BNMA using a random-effects JAGS model, with SMD as the primary outcome measure. Compared with CON, AUBI showed the largest improvement in sleep quality (SMD = −1.02; 95% CrI: −1.43 to −0.60), followed by OBI (SMD = −0.56; 95% CrI: −0.96 to −0.16), MBI (SMD = −0.54; 95% CrI: −0.88 to −0.19), and PABI (SMD = −0.49; 95% CrI: −0.71 to −0.27), all of which demonstrated statistically significant effects relative to the control condition. LBI was associated with a modest but borderline significant improvement (SMD = −0.41; 95% CrI: −0.81 to −0.01), whereas RBI did not differ significantly from CON (SMD = −0.25; 95% CrI: −0.65 to 0.15). The relative effects between intervention categories are summarized in Figure 4. While point estimates suggested potential differences between active NBI modalities, the vast majority of pairwise comparisons between intervention categories had wide 95% CrIs that included the null value of zero. This indicates that no single NBI modality demonstrated clear superiority over any other.

### Treatment ranking based on SUCRA

**Figure 5:**
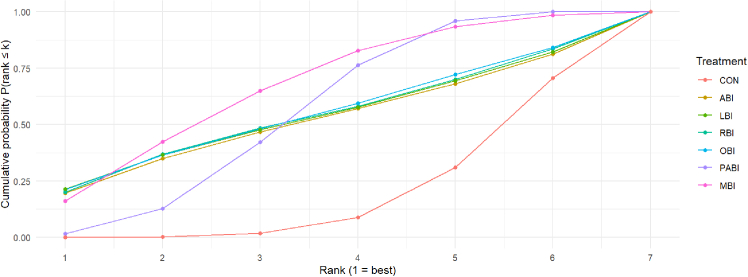
Treatment ranking probabilities based on Bayesian network meta-analysis Cumulative ranking probability curves for each intervention, derived from the Bayesian network meta-analysis. The surface under the cumulative ranking curve (SUCRA) reflects the probability that each intervention ranks among the most effective for improving sleep quality, with higher SUCRA values indicating greater effectiveness. Abbreviations: PABI, physical activity-based interventions; AUBI, auditory-based interventions; OBI, olfactory-based interventions; LBI, light-based interventions; RBI, respiratory-based interventions; MBI, multisensory-based interventions; CON, conventional care.

Treatment ranking probabilities were summarized using cumulative ranking curves and the surface under the cumulative ranking curve (SUCRA), derived from PSQI outcomes (where lower PSQI scores indicate better sleep quality; Figure 5). MBI and PABI consistently demonstrated the highest ranking probabilities, with cumulative curves rapidly ascending within the top ranks, indicating a high likelihood of being among the most effective interventions. Conversely, single-modality interventions, comprising AUBI, OBI, LBI, and RBI, exhibited intermediate ranking profiles with largely overlapping cumulative curves, indicating comparable yet less pronounced benefits. CON received consistently low rankings, with a majority of its cumulative probability concentrated in the worst ranks. The SUCRA results indicate a clear hierarchical pattern, with MBI and PABI outperforming single-modality NBIs and CON in terms of probabilistic treatment ranking.

### Sensitivity analyses

The leave-one-study-out (LOO) sensitivity analysis demonstrated that the BNMA results were highly robust. Across all iterations, both the magnitude of treatment effects and the relative ordering of interventions remained stable. AUBI consistently showed the largest improvement in sleep quality, with SMDs ranging approximately from −0.98 to −1.07 across iterations. OBI, MBI, and PABI demonstrated moderate and stable benefits, with SMD ranges of −0.52 to −0.61 for OBI, −0.50 to −0.58 for MBI, and −0.45 to −0.52 for PABI, respectively. LBI exhibited smaller and more variable effects, with SMDs ranging from −0.36 to −0.44, whereas RBI consistently showed little to no effect, with estimates fluctuating around the null value and CrIs overlapping zero. Importantly, the ranking of intervention categories remained nearly identical to that observed in the primary analysis, indicating that no single study exerted a disproportionate influence on the network estimates or overall ranking structure. These findings confirm the stability of the main results. Complete numerical outputs and iteration-level estimates from the LOO analyses are provided in the supplemental information.

**Figure 6:**
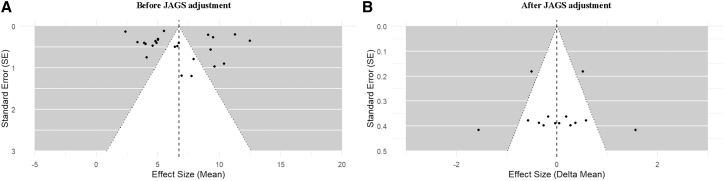
Funnel plots assessing publication bias before and after Bayesian adjustment Comparison-adjusted funnel plots evaluating potential small-study effects and publication bias in the network meta-analysis. (A) Funnel plot of effect estimates against their standard errors prior to Bayesian adjustment, showing moderate asymmetry among comparisons with larger standard errors. (B) Funnel plot after Bayesian random-effects adjustment, showing improved symmetry of effect estimates around the null-centered reference line, indicating a reduced influence of small-study effects.

To further assess the robustness of the BNMA results and the potential impact of small-study effects, comparison-adjusted funnel plots were examined before and after Bayesian model adjustment (Figure 6). Prior to the implementation of JAGS adjustment, the funnel plot exhibited moderate asymmetry, particularly among comparisons with larger standard errors, suggesting the potential presence of small-study effects or between-study heterogeneity. Subsequent to implementing the Bayesian random-effects adjustment, the funnel plot exhibited enhanced symmetry around the null-centered reference line, with effect estimates demonstrating increased tight clustering and an absence of discernible directional imbalance across standard errors. In conjunction with the LOO analyses, these findings suggest that the primary BNMA outcomes were not influenced by small-study effects or individual influential studies, thereby substantiating the overall robustness of the network estimates.

### Assessment of network consistency

The global consistency was assessed using the Bayesian consistency model for the MBI-PABI comparison. The posterior mean difference was −1.09, with a 95% CrI from −4.38 to 2.21. The CrI included the null value, indicating no evidence of global inconsistency at the network level. The model convergence was satisfactory, with potential scale reduction factors close to unity and large effective sample sizes across all parameters. Local consistency was evaluated using a Bayesian node-splitting analysis. The direct estimate yielded a posterior mean of −0.68 with a 95% CrI from −1.88 to 0.55, while the indirect estimate showed a posterior mean of −2.59 with a 95% CrI from −8.59 to 3.34. The difference between direct and indirect estimates had a posterior mean of 1.93, with a 95% CrI spanning −4.11 to 8.07 and a Bayesian two-sided posterior probability of 0.53, indicating no evidence of inconsistency between direct and indirect evidence (Table 2).

**Table 2:** Node-splitting results for the MBI vs. PABI comparison

Estimate (MBI-PABI)	Mean	SD	95% CrI (lower)	95% CrI (upper)	Bayesian *p*-value
Direct	−0.68	0.62	−1.88	0.55	–
Indirect	−2.59	3.03	−8.59	3.34	–
Direct – Indirect	1.93	3.10	−4.11	8.07	0.53

“direct,” posterior estimate from the model using only direct evidence; “indirect,” posterior estimate from the model using only indirect evidence; “direct-indirect,” difference between direct and indirect estimates; SD, posterior standard deviation; CrI, credible interval. All estimates are reported on the same scale as the primary network model. Bayesian *p*-value refers to the two-sided posterior probability of inconsistency. Convergence diagnostics for all models were satisfactory ($\hat{R}$≈1.00, large effective sample size [n.eff]). The 95% CrI for all estimates includes the null value of zero, indicating no evidence of statistically significant inconsistency.

## Discussion

From an evidence-mapping perspective, the present BNMA reflects a salient characteristic of the extant literature on NBIs for sleep: Despite the mounting research interest, the number of RCTs that meet strict eligibility criteria remains limited. The distribution of eligible studies over time was uneven, with the majority of trials published after 2020 and a notable concentration in the post-pandemic period, particularly around 2023. Conversely, the number of high-quality RCTs published in 2024–2025 has been limited. Recent systematic reviews of non-pharmacological sleep interventions have documented analogous temporal patterns, a phenomenon that is presumably attributable to the practical and ethical challenges associated with conducting controlled nature-based trials, as opposed to a waning of scientific interest. In light of the current state of the evidence base, which is characterized by its modest development and fragmentation, the BNMA framework offers a valuable approach for integrating these disparate pieces of evidence and facilitating cautious comparative inferences.

In light of these findings, the BNMA identified disparities in effectiveness across NBI categories, while concurrently demonstrating a consistent and substantial overall benefit of NBIs in comparison to CON. A range of intervention categories, including AUBI, OBI, MBI, and PABI, exhibited moderate and clinically significant enhancements in PSQI scores relative to CON. Although AUBI was positioned toward the upper end of the scale, the magnitude of its estimated effect was analogous to that of other active NBIs, with overlapping uncertainty intervals across most pairwise comparisons. These results suggest that, rather than a single modality that is clearly dominant, multiple forms of sensory-based NBIs offer broadly similar benefits for sleep quality.

This pattern aligns with the findings reported in the extant literature. Previous meta-analyses and RCTs have shown that AUBI, particularly music therapy, is associated with significant reductions in PSQI scores relative to control groups.^117^^,^^118^^,^^119^ However, the effect sizes observed are typically comparable to those reported for aromatherapy and structured physical activity. Similarly, OBI, particularly the inhalation of essential oils such as lavender, has been associated with moderate improvements in global PSQI scores across numerous trials and systematic reviews.^120^^,^^121^ Existing literature indicates that MBI, including forest therapy and horticultural therapy, may exert beneficial effects on both subjective and objective sleep outcomes.^70^^,^^72^^,^^122^^,^^123^ However, the paucity of eligible RCTs limits the ability to make definitive comparisons. The efficacy of PABI is supported by a relatively extensive evidence base, which consistently demonstrates moderate improvements in PSQI scores when compared with no-exercise controls.^124^^,^^125^^,^^126^ In contrast, LBI has been found to produce more modest changes in subjective sleep quality, with more consistent effects reported for sleep continuity and circadian alignment.^127^^,^^128^ However, the evidence for RBI remains heterogeneous across diverse populations and settings.^129^^,^^130^

A synthesis of the BNMA results and the broader literature suggests a convergent conclusion: NBI categories consistently exhibit greater efficacy in improving sleep quality compared with CON. However, it is imperative to exercise caution when interpreting these findings, as the differences observed among specific NBI categories are often modest in nature. While the included trials showed robust performance in random sequence generation and outcome reporting, the high prevalence of performance bias, along with residual concerns regarding allocation concealment and other bias domains, warrants cautious interpretation of the pooled findings. The observed overlap in effect estimates suggests the presence of multiple sensory and experiential pathways that may be equally capable of facilitating sleep improvement. From a clinical and public health perspective, this suggests that the selection of NBI modalities may be reasonably guided by factors such as feasibility, individual preference, and contextual factors, rather than by minor variations in comparative ranking. Concurrently, these findings underscore the necessity for additional, methodologically robust, head-to-head RCTs of a larger scale to further elucidate the relative effectiveness across sensory-based NBI categories. These interpretive insights should be considered alongside the robustness of the network evidence, which provides an important basis for assessing the reliability of the comparative conclusions.

First, the LOO sensitivity analysis is a crucial robustness check for the comparative conclusions drawn from a relatively small and heterogeneous RCT evidence base.^131^ In principle, LOO addresses the question of whether network estimates are excessively dependent on a single trial that might differ in population characteristics, intervention implementation, or risk-of-bias profile. The stability observed across LOO iterations suggests that the principal conclusions are supported by the broader structure of the evidence rather than being anchored to a single influential study. This is of particular pertinence for sensory-classified NBIs, wherein certain categories are represented by a limited number of trials. In such settings, an apparent “best” intervention can, at times, be indicative of idiosyncratic study features rather than a reproducible effect. In accordance with these findings, the LOO findings provide a high degree of confidence in the direction of the primary conclusion, which is that active NBI categories tend to improve PSQI relative to CON. However, the findings also underscore the necessity of interpreting fine-grained ranking differences with caution when effect estimates remain close, and uncertainty intersects across interventions.

Second, the comparison-adjusted funnel plots complement LOO by evaluating the extent to which small-study effects or selective reporting could distort network-level inferences.^132^ According to the findings of this study, the implementation fidelity, participant adherence, and outcome assessment of smaller trials are often subject to variability. These trials may also be more susceptible to publication bias, a factor that has the potential to engender asymmetry in unadjusted effect distributions. The enhanced symmetry that emerges following model-based adjustment should be interpreted as indicative of the primary conclusions not being predominantly driven by a small subset of imprecise studies with extreme estimates. It is important to note that this does not imply that publication bias has been “eliminated.” Rather, it suggests that, under the modeling assumptions, the synthesis is less sensitive to imprecision-related dispersion and the influence of small trials is moderated. In practical terms, this lends credence to the network-level inference that NBIs, considered as a group, are advantageous in comparison with CON, while concurrently upholding a prudently conservative posture with respect to the precise magnitude disparities among specific NBI categories.

Third, the consistency and inconsistency assessments serve to elucidate whether the network evidence is coherent across direct and indirect pathways. In networks where numerous comparisons depend on indirect evidence, the absence of substantial discrepancy between direct and indirect estimates offers assurance that the transitivity and consistency assumptions are not evidently violated for the salient contrasts examined. Consequently, the node-splitting results function as an interpretive safeguard, indicating that the comparative conclusions are not being generated by conflicting evidence streams that would undermine causal interpretability at the network level.^133^ Concurrently, the value of consistency diagnostics in this review should be considered alongside the structural constraints of the evidence base—namely, sparse connections for certain categories and limited numbers of head-to-head trials. Consequently, while the extant diagnostics support internal coherence, they also underscore that future research priorities should include more direct comparative RCTs between leading sensory-based NBI categories. These RCTs would increase the precision of relative effects and reduce reliance on indirect comparisons for ranking.

Meanwhile, these findings highlight several priorities for future research. Future research should prioritize conducting larger, well-designed RCTs and expanding the representation of currently underpopulated comparison nodes to increase the robustness and interpretability of network estimates. Although Bayesian modeling helps mitigate this limitation by incorporating both direct and indirect evidence, a larger dataset would further improve the accuracy and reliability of the estimated treatment effects. Future research should therefore prioritize large-scale, rigorously designed RCTs with standardized outcome reporting and longer follow-up periods to clarify the durability of NBI effects on sleep health. Particular attention should be paid to elucidating dose-response relationships and identifying optimal combinations of sensory modalities, intervention intensity, and exposure duration. Incorporating objective sleep measures, such as actigraphy, polysomnography, and physiological biomarkers related to autonomic and circadian regulation, would further strengthen causal inference and mechanistic understanding. Finally, expanding research to more diverse populations and real-world settings will be essential for translating evidence on NBIs into precise, scalable, and equitable public health strategies for improving sleep health.

These considerations collectively highlight the broader implications of the present synthesis for both research and practice. This BNMA offers a comparative synthesis of randomized evidence concerning the effects of NBIs on adult sleep health. Across intervention categories, nature-based approaches demonstrated consistent superiority over CON, while differences between specific modalities were generally modest, suggesting a broadly shared therapeutic benefit of nature-oriented strategies rather than a reliance on a single dominant approach. Taken together, these findings support the role of non-pharmacological, sensory-oriented interventions as effective and scalable options for sleep health promotion. At the same time, they point to the importance of future work focused on larger, methodologically rigorous trials and integrative intervention designs to refine comparative effectiveness estimates and further strengthen the evidence base for public health implementation.

### Limitations of the study

Although this BNMA synthesized the currently available randomized evidence, the overall number of eligible RCTs remained limited after applying strict inclusion criteria, particularly those requiring PSQI as a unified outcome measure. The evidence base comprised only 13 RCTs, resulting in a relatively sparse treatment network in which several comparisons were informed by single studies. This structural sparsity contributed to unstable posterior estimates and wide CrIs for multiple contrasts, thereby limiting the precision of the comparative estimates. Sparse networks inherently constrain the stability of indirect comparisons and restrict the borrowing of strength across nodes, meaning that some findings should be interpreted as preliminary and with caution. These constraints reflect both the methodological heterogeneity of the existing literature and the practical challenges associated with conducting controlled nature-based trials, particularly for less frequently studied intervention categories.

Beyond the limited size of the evidence base, methodological limitations within individual trials may also have influenced the interpretation of the findings. Several studies had short follow-up periods or insufficient methodological reporting, including unclear randomization procedures and allocation concealment, increasing the risk of bias. Consistent with the RoB 2 assessment, some comparisons were rated as low or very low in methodological quality, and incomplete reporting further limited interpretability. Differences in sample size, baseline sleep characteristics, and study settings also contributed to between-study variability.

In addition, although categorizing NBIs by their primary sensory or experiential modality enhanced conceptual clarity, considerable within-category heterogeneity remained in intervention intensity, duration, delivery format, and environmental context. Such variability may obscure dose-response relationships and attenuate differences between intervention categories. Moreover, reliance on PSQI as a single outcome metric precluded a more granular assessment of specific sleep dimensions, including sleep efficiency, architecture, and circadian timing, which may respond differently to distinct sensory pathways. Most included trials also assessed short-to medium-term outcomes, leaving uncertainty regarding the long-term sustainability of observed benefits and the optimal frequency of intervention delivery. Taken together, these factors limit the precision, interpretability, and generalizability of the present findings.

## Resource availability

### Lead contact

Further information and requests for resources and materials should be directed to and will be fulfilled by the Lead Contacts, Qibing Chen (cqb@sicau.edu.cn).

### Materials availability

This study did not generate new materials.

### Data and code availability

• This paper analyzes existing, publicly available data derived from published randomized controlled trials. The extracted dataset supporting the findings of this study is provided in the supplemental information and is publicly available as of the date of publication.
• All original code used for data processing and Bayesian network meta-analysis has been deposited at Zenodo (https://doi.org/10.5281/zenodo.18731498) and is publicly available as of the date of publication.
• Any additional information required to reanalyze the data reported in this paper is available from the lead contact upon request.

## Acknowledgments

This work was partially supported by the 10.13039/501100001809National Natural Science Foundation of China, China (3227l40499), the Subproject of the 10.13039/501100012166National Key Research and Development Program of the 14th Five-Year Plan for Multi-Objective Precision and Efficient Cultivation Technologies for Bamboo and Rattan Resources, China (2023YFD2201204), the Chengdu Science and Technology Talent Support Program, China (2024-RC02-00010-CG), and Doctoral Training Grants from 10.13039/501100008363Sichuan Agricultural University, China. We sincerely thank all institutions and researchers who contributed to and supported this work.

## Author contributions

Conceptualization, C.M. and S.L.; methodology, C.M. and A.M.S.; software, C.M., W.C., Z.L., and R.W.; validation, C.M.; formal analysis, C.M., A.M.S., and W.C.; investigation, Z.L., R.W., and X.D.; resources, B.L. and Q.C.; data curation, C.M., A.M.S., Z.L., Y.P., and X.D.; writing – original draft, C.M. and S.L.; writing – review and editing, A.M.S., H.L., B.L., S.L., and Q.C.; visualization, C.M., W.C., Y.P., and H.L.; supervision, S.L. and Q.C.; project administration, Q.C.; funding acquisition, S.L. and Q.C.

## Declaration of interests

The authors declare no competing interests.

## Declaration of generative AI and AI-assisted technologies in the writing process

During the preparation of this work, the authors used ChatGPT (OpenAI) to assist with language polishing and editorial improvements. All scientific content, interpretations, and conclusions were developed by the authors, who reviewed and take full responsibility for the final manuscript.

## STAR★Methods

### Key resources table

**Table:** 

REAGENT or RESOURCE	SOURCE	IDENTIFIER
**Deposited data**		

Extracted RCT dataset for Bayesian network meta-analysis	Included published randomized controlled trials	Table S1
Full analysis code for Bayesian network meta-analysis	Zenodo	https://doi.org/10.5281/zenodo.18731498

**Software and algorithms**		

R statistical computing environment	R Foundation for Statistical Computing	Version 4.5.2
R2jags package	R Foundation for Statistical Computing	Version 1.1-01
netmeta package	R Foundation for Statistical Computing	Latest version
ggplot2 package	R Foundation for Statistical Computing	Latest version
EndNote	Clarivate	Version 21.2
Review Manager (RevMan)	Cochrane Collaboration	Version 5.3
Just Another Gibbs Sampler (JAGS)	JAGS Development Team	Version 4.3.1

**Other**		

Cochrane Risk of Bias 2 (RoB 2) Tool	Cochrane Collaboration	https://www.riskofbias.info/
PRISMA 2020 Guidelines	EQUATOR Network	https://www.equator-network.org/reporting-guidelines/prisma/
PROSPERO Registration	International Prospective Register of Systematic Reviews	Registration No. CRD420251059058

### Experimental model and study participant details

This study did not recruit new human participants or experimental animal models. Instead, it synthesized aggregated data from previously published randomized controlled trials investigating nature-based interventions for sleep outcomes. All participant-level characteristics (including age, sex, and health status) were extracted directly from the original published trials. Ethical approval and informed consent were obtained by the investigative teams of the original trials; therefore, no additional institutional review board approval was required for this secondary analysis. Full details of participant demographics and study characteristics are summarized in Table 1. Because this study relied solely on aggregated published data, formal analyses of sex or gender differences across interventions could not be conducted, which may limit the generalizability of the findings.

### Method details

#### Literature search approach

We systematically searched five electronic databases: Web of Science (WoS), Cochrane Library, Embase, ScienceDirect, and PubMed, from database inception to October 31, 2025. The full search strategy for PubMed is presented below; search operators were adjusted slightly for each database to align with platform-specific syntax.

PubMed search strategy: ((“Lighting therapy” OR “Sunlight exposure” OR “Physical exercise therapy” OR “Physical activity” OR “Air therapy” OR “Air exposure” OR “Forest therapy” OR “Forest bathing” OR “Horticulture therapy” OR “Fragrance therapy” OR “Aroma therapy” OR “Music therapy” OR “Sound exposure” OR “Light therapy”[Mesh] OR “Sunlight”[Mesh] OR “Exercise Therapy”[Mesh] OR “Exercise”[Mesh] OR “Forest Bathing”[Mesh] OR “Horticultural Therapy”[Mesh] OR “Aromatherapy”[Mesh] OR “Music Therapy”[Mesh]) AND (“sleep health” OR “sleep quality” OR “sleep disorder∗” OR “Sleep”[Mesh] OR “Sleep Wake Disorders”[Mesh])∗∗.

#### Eligibility criteria

Duplicate records were identified and removed using EndNote 21.2. Two independent reviewers screened all titles and abstracts for eligibility; any discrepancies were resolved via consensus consultation with a third senior reviewer. Full-text articles were then assessed against pre-specified inclusion and exclusion criteria. The study selection process was documented in accordance with the Preferred Reporting Items for Systematic Reviews and Meta-Analyses (PRISMA) 2020 guidelines, using a standardized flow diagram. Eligibility criteria were rigorously defined using the Participants, Interventions, Comparisons, Outcomes, Study design (PICOS) framework.^134^

Inclusion criteria were: (1) Participants: Adults with diagnosed sleep disorders, self-reported sleep disturbances, or poor sleep quality; (2) Interventions: Studies evaluating nature-based interventions (NBIs) categorized by their primary sensory or experiential modality, including forest therapy, light therapy, aromatherapy, sound/music therapy, air therapy, forest walking, nature walks, green exercise programs, horticultural therapy, and programs combining physical activity with natural environment exposure; (3) Comparators: Standard treatment, usual care, wait-list controls, or baseline conditions without NBI exposure; (4) Outcomes: Studies reporting sleep outcomes assessed using the validated Pittsburgh Sleep Quality Index (PSQI), including either global PSQI scores or validated component scores; (5) Study design: Only randomized controlled trials (RCTs) were included.

Exclusion criteria were: (1) Studies focusing on specific clinical or occupational populations, including pregnant or postpartum women; perioperative patients or those with severe acute illness; individuals with cancer or other malignant diseases; shift workers or those with atypical sleep schedules; participants with neurological or cognitive disorders; populations with metabolic, endocrine, or cardiovascular disease; and perimenopausal/menopausal women. Studies including infants, children, or adolescents were also excluded; (2) Studies evaluating interventions combining NBIs with pharmacological treatments, where the independent effect of NBIs could not be isolated; (3) Animal studies, *in vitro* studies, case reports, case series, reviews, editorials, and qualitative studies; (4) RCTs with excessive participant dropout (>20%, per Cochrane Handbook guidelines); (5) Studies with critical statistical errors or insufficient data to calculate effect sizes; (6) Studies not published in English; (7) Studies where the primary outcome was not related to sleep (e.g., studies focusing solely on stress, affect, mood, exercise adherence, self-esteem, or other non-sleep outcomes).

#### Data collection process

The study selection process for this BNMA is summarized in detail in the figure below. The initial search across five databases identified 20,551 records. After duplicate removal, 15,739 records advanced to the title and abstract screening stage. At this stage, two independent reviewers screened all records against predefined eligibility criteria, resulting in the exclusion of 15,526 records. The remaining 213 articles underwent full-text eligibility assessment. After full-text review, 200 studies were excluded due to methodological ineligibility, inappropriate populations or interventions, or insufficient/unavailable sleep-related outcome data. Ultimately, 13 RCTs met all inclusion criteria and were included in both qualitative and quantitative analyses.

**Figure:**
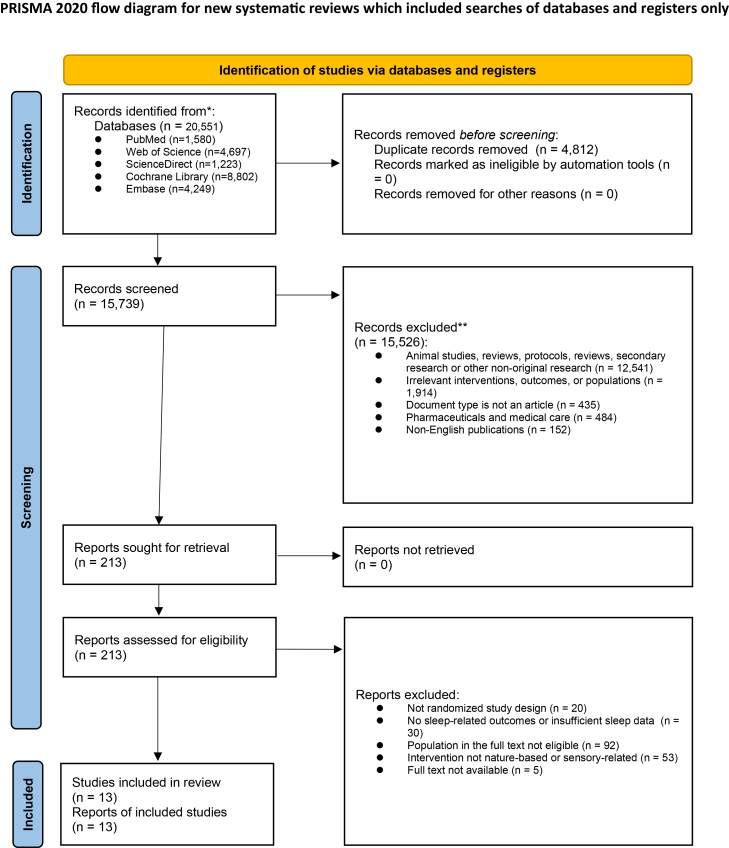
PRISMA 2020 flow diagram of study selection This flow diagram summarizes the study identification, screening, eligibility assessment, and final inclusion process, conducted in accordance with the Preferred Reporting Items for Systematic Reviews and Meta-Analyses (PRISMA) 2020 guidelines. The number of records at each stage and the reasons for exclusion are detailed. This flow diagram follows the PRISMA 2020 format for new systematic reviews, including searches of databases and clinical trial registers only.

Data extraction was conducted independently by two reviewers using a pre-specified standardized data extraction form. All reviewers independently verified the research design, participant characteristics, sample sizes, NBI details, intervention and control group specifications, and outcome measurement protocols of all included studies. Discrepancies between reviewers were resolved via consensus discussion, with arbitration by a third senior reviewer if required.

#### Assessment of study bias

We exclusively included RCTs, and assessed the methodological quality of each included trial using the Cochrane Risk of Bias 2 (RoB 2) tool, implemented in Review Manager 5.3.^135^^,^^136^

### Quantification and statistical analysis

We conducted a Bayesian network meta-analysis (BNMA) using the R2jags package (version 1.1-01) in R (version 4.5.2). Parameter estimation was performed via Markov Chain Monte Carlo (MCMC) sampling, implemented in Just Another Gibbs Sampler (JAGS, version 4.3.1). We ran 3 independent MCMC chains, and assessed model convergence using the Gelman-Rubin potential scale reduction factor ($\hat{R}$), with $\hat{R}$ < 1.05 indicating satisfactory convergence. Model stability and robustness were further verified via multiple diagnostic checks.^137^^,^^138^^,^^139^^,^^140^^,^^141^ To assess the robustness of the primary BNMA findings, we conducted a leave-one-study-out (LOO) sensitivity analysis. The BNMA model was re-estimated iteratively, with one trial removed in each iteration. For each iteration, we extracted the posterior mean treatment effect for each intervention versus the control group, and compared these estimates to those from the primary analysis. All findings are reported as effect sizes with corresponding 95% credible intervals (CrIs).

Furthermore, given the presence of a closed loop in the treatment network, we formally assessed the consistency assumption of the BNMA. Local inconsistency was evaluated using a Bayesian node-splitting approach, in which direct and indirect evidence for the target comparison were separated and modeled independently. For the comparison informed by a single direct trial, the direct estimate was analyzed using a fixed-effect Bayesian model, while the indirect estimate was derived from a random-effects consistency network meta-analysis excluding the direct trial data. Inconsistency was quantified as the posterior distribution of the difference between direct and indirect estimates. Evidence of inconsistency was assessed based on whether the 95% CrI of this difference excluded the null value of zero, in conjunction with the two-sided Bayesian posterior probability of inconsistency. Model convergence for all node-splitting analyses was evaluated using the potential scale reduction factor ($\hat{R}$) and the effective sample size (n.eff) from the Bayesian sampling output. We calculated the surface under the cumulative ranking curve (SUCRA) using the nma.rank() function to generate hierarchical rankings of all interventions. League tables of relative treatment effects were generated using the nma.league() function. SUCRA curves, ranking heatmaps, and forest plots were visualized using the ggplot2 and netmeta packages in R. To assess potential publication bias and small-study effects, we generated comparison-adjusted funnel plots, both before and after adjustment via the Bayesian JAGS model.^142^^,^^143^^,^^144^

### Additional resources

This systematic review and network meta-analysis was prospectively registered in the International Prospective Register of Systematic Reviews (PROSPERO) under registration number CRD420251059058. The full registration record is available at: https://www.crd.york.ac.uk/PROSPERO/view/CRD420251059058.
